# Impact of multimorbidity on healthcare costs in patients with type 2 diabetes in China: a longitudinal analysis of health insurance claims data

**DOI:** 10.1186/s13690-025-01746-6

**Published:** 2025-11-06

**Authors:** Xing Chen, Luying Zhang, Wen Chen

**Affiliations:** 1 Shanghai Institute of Infectious Disease and Biosecurity, Fudan University, Shanghai, China; 2 School of Public Health, Fudan University, Shanghai, China

**Keywords:** Diabetes, Multimorbidity, Claims data, Prevalence, Healthcare cost

## Abstract

**Background:**

Multimorbidity is highly prevalent among individuals with diabetes and exerts a substantial impact on healthcare systems. This study aims to investigate the prevalence and healthcare costs of multimorbidity in patients with type 2 diabetes and to assess the influence of multimorbidity on healthcare expenditures using machine learning approaches.

**Methods:**

We conducted a retrospective cohort study utilizing chronic disease management database and health insurance claim database from a city in eastern China. Twenty-nine multimorbidities with a prevalence exceeding 1% among diabetic patients were identified using ICD codes. We analyzed the trends in prevalence and healthcare costs from 2014 to 2019. Machine learning models were developed to predict healthcare expenditures, and SHAP analysis was applied to the optimal model to evaluate the contribution of specific multimorbidity to healthcare costs.

**Results:**

Among 79,910 patients, the prevalence of multimorbidity increased from 87.9% in 2014 to 99.3% in 2019, while the proportion of healthcare costs attributed to multimorbidity rose from 31.8% to 34.2%. In 2019, the most prevalent conditions were hypertension (88.3%), arthritis (74.7%), and chronic ischemic heart disease (54.6%), whereas the highest-cost conditions included sequelae of cerebrovascular disease ($3,860.8), cerebral infarction ($2,768.8), and renal failure ($1,543.9). SHAP analysis revealed that cerebrovascular disease sequelae, heart failure, chronic ischemic heart disease, and chronic obstructive pulmonary disease had the most significant impact on future healthcare costs for diabetic patients.

**Conclusions:**

Multimorbidity is nearly universal among individuals with diabetes in China, with cardiovascular, cerebrovascular, and chronic respiratory diseases contributing disproportionately to healthcare expenditures.

**Supplementary Information:**

The online version contains supplementary material available at 10.1186/s13690-025-01746-6.

**Table:** 

Text box 1. Contributions to the literature	
• China, with many diabetic patients, lacks comprehensive data on diabetes multimorbidity burden and costs due to limited past studies. • High diabetes multimorbidity rates and costs emphasize the need for early screening and management. • Key conditions like cerebrovascular disease sequelae, heart failure, chronic ischemic heart disease and chronic obstructive pulmonary disease drive costs, requiring targeted interventions.	

## Introduction

Diabetes mellitus is a chronic disease characterized by impaired glucose homeostasis and ranks as the eighth leading cause of death and disability globally [1, 2]. According to the Global Burden of Disease 2021 study, approximately 529 million people worldwide have diabetes, with an age-standardized prevalence of 6.1% [3]. The rising prevalence of diabetes is also placing a heavy burden on the global health system, with the International Diabetes Federation estimating that global health expenditures for diabetes-related medical costs amount to $966 billion in 2021 [4]. China has one of the heaviest burdens of diabetes, with the prevalence of diabetes expected to increase from 8.2% in 2020 to 9.7% in 2030, and the total cost of diabetes projected to increase from $250.2 billion to $460.4 billion [5].

It’s been reported that 77%−90% of patients with type 2 diabetes have one or more multimorbidities, such as cardiovascular disease, diabetic kidney disease, diabetic retinopathy, diabetic neuropathy, and many others [6, 7]. Multimorbidity in patients with diabetes mellitus complicates treatment and management, leading to various adverse outcomes, including decreased quality of life, more hospitalizations, and premature death [8]. More importantly, multimorbidity significantly impacts healthcare costs for people with diabetes, not just in the year in when an event occurs, but by permanently increasing the average level of inpatient and non-inpatient costs in subsequent years [9].

Recent years have seen an increase in the use of health insurance claims data to study the impact of multimorbidity on the economic burden of patients with diabetes. Examples include identifying the direct medical costs of multimorbidities such as microvascular complications and macrovascular complications, analyzing the impact of the number of multimorbidities on healthcare costs, and using latent categories to analyze the clinical characteristics of high-cost patients with diabetes [10–12]. However, most studies in China on the burden of diabetes multimorbidity have used cross-sectional study designs and data from only a few hospitals [13–15]. Meanwhile, when exploring the relationship between multimorbidity and healthcare costs, existing research have focused on the impact of multimorbidity numbers and on describing healthcare costs resulting from specific multimorbidity or combinations of multimorbidity [16–18]. Therefore, a more comprehensive understanding of the burden and economic impact of multimorbidity in Chinese diabetes is essential for providing valuable information to clinicians and policymakers for intervention development.

This study will use longitudinal health insurance claims data from a large city in China to identify diabetic patients with multimorbidity based on their actual medical visits. The goal is to describe changes in the burden of diabetic multimorbidity and to determine the impact of multimorbidity on healthcare costs based on machine learning models.

## Research design and methods

### Study setting

Multimorbidity is becoming increasingly prevalent among people with diabetes and causes a significant economic burden. Research on the burden of diabetes multimorbidity in low-income and middle-income countries are lacking. The Chinese government has released the Diabetes Prevention and Control Action Implementation Plan 2024–2030, which proposes to strengthen the screening and intervention management of diabetes multimorbidity.

### Data sources

The data for this study was obtained from the Health Insurance Claim Database and the Chronic Disease Management Database of a large city in eastern China. The Health Insurance Claim Database contains demographic information, consultation details, and cost information of the city’s Urban Employee Basic Medical Insurance (UEBMI) and Urban and Rural Residents Basic Medical Insurance (URRBMI) participants. The Chronic Disease Management Database contains demographic information, complications, comorbidities, and physical indicators for diabetic and hypertensive patients in the city. After signing a confidentiality agreement, we were provided with chronic disease health management data and health insurance claims data for the sample city from 2014 to 2019 by the sample city’s Health Security Administration and Health Commission, all of which concealed personally identifiable information.

### Sample population

**Fig. 1:**
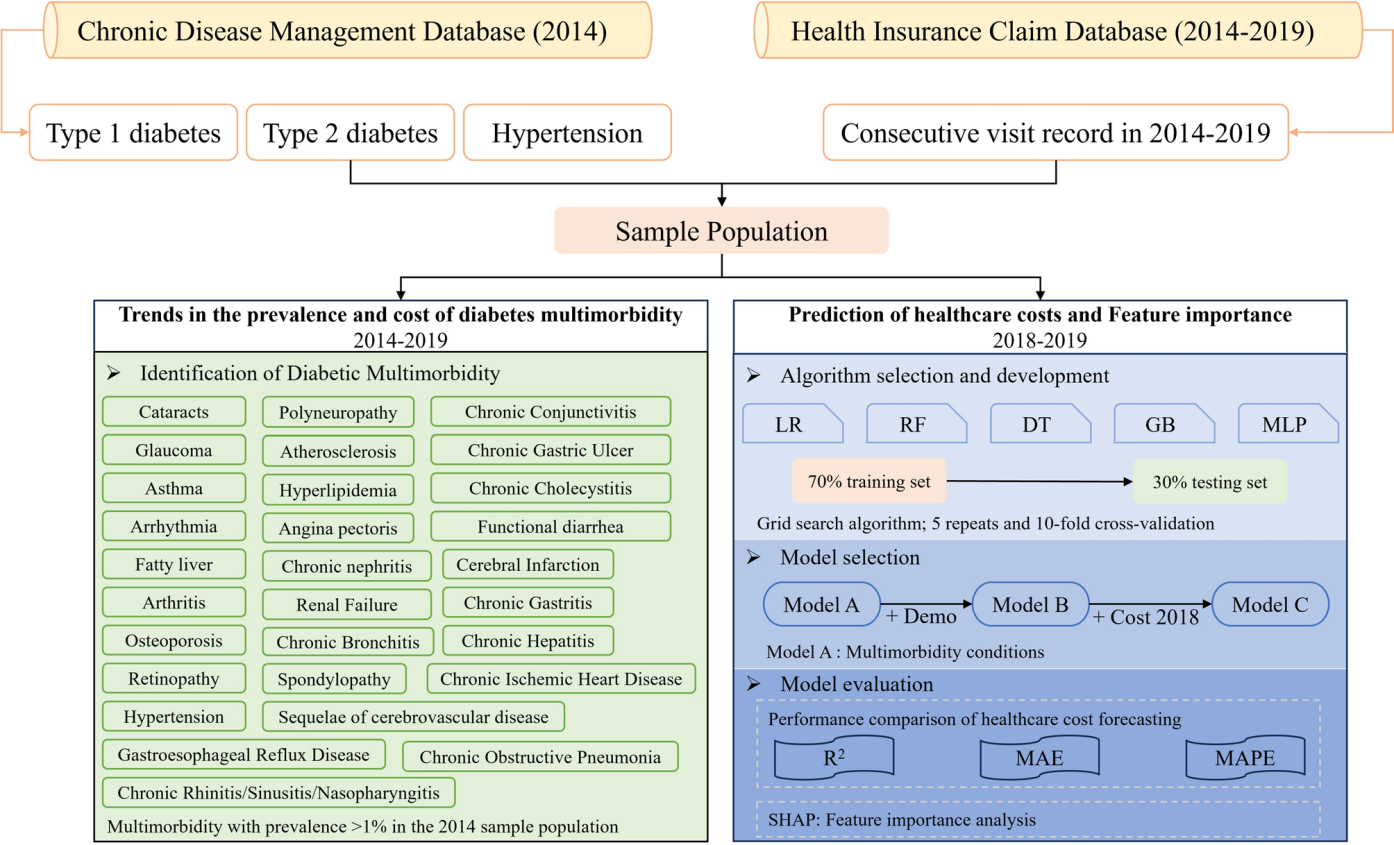
Study flowchart of impact of multimorbidity on healthcare costs among diabetic patients in China, 2014–2019. LR: linear regression, RF: random forest, DT: decision tree, GB: gradient boosting, MLP: multilayer perceptron, MAE: mean absolute error, MAPE: mean absolute prediction error

At first, we selected all cases registered with type 2 diabetes (ICD E11) from the Chronic Disease Management Database in 2014. At the same time, a longitudinal cohort of individuals with continuous medical visit records from 2014 to 2019 was identified in the Health Insurance Claims Database. Then, we connected the Chronic Disease Management Database with the Health Insurance Claim Database by the patient’s unique identifier. Finally, we included all patients with type 2 diabetes who were registered in the Chronic Disease Management Database in 2014 and had consecutive visits from 2014 to 2019 (Fig. 1). We extracted data on patients’ gender, age, height, weight, insurance type, diagnosis of visit, and healthcare costs as study variables.

### Identification of diabetes multimorbidity

In the sample population, multimorbidity condition was defined as having at least one outpatient or inpatient ICD-10 diagnosis recorded in the current year. Also, patients registered in the Chronic Disease Management Database at baseline for comorbidities and complications were included in the analysis. Furthermore, we believe that even if a patient does not have a medical record of the corresponding disease in the second year, he will continue to suffer from the said disease in the following years. In this study, 29 somatic-related multimorbidities were identified by ICD codes among diabetic patients (Fig. 1). These multimorbidities were selected based on literature studies and had an actual occurrence of more than 1% among type 2 diabetes patients in the sample city in 2014.

### Healthcare costs

We examined the yearly healthcare costs and costs associated with specific multimorbidity conditions for our sample population. Yearly healthcare costs included the total of patients’ outpatient visits, inpatient hospital stays, and pharmacy purchases throughout the year. The costs related to specific multimorbidities were the expenses incurred by patients for medical procedures related to those specific conditions (identified by ICD-10 codes). Additionally, we calculated the annual per capita medical costs for multimorbidity based on the number of patients. During the analysis, we excluded visits record with unusually high or negative anomalies in medical costs. Ultimately, we obtained annual medical costs and medical costs attributable to multimorbidity for all diabetic patients included in the study from 2014 to 2019.

### Statistical analysis

The study used Stata 17.0 to process the data to identify the sample population and calculate the prevalence of multimorbidity and healthcare costs. Additionally, the study developed predictive models using different machine learning algorithms constructed to include different variables for predicting patients’ healthcare costs in 2019. Machine learning methods are particularly useful for modeling the complex, non-linear relationships and interactions between multimorbidity and patient healthcare costs in large populations, which traditional regression models may fail to adequately capture. By applying machine learning algorithms, it is possible not only to build well-performing cost prediction models but also to identify important multimorbidity features associated with healthcare expenditures in a data-driven manner, thereby informing targeted clinical and policy interventions. The sample population was split into two datasets: a training set and a testing set. In the training set, 70% of diabetes cases were randomly selected. The remaining sample was assigned to the testing set. One reference algorithm and four machine learning algorithms were developed using the training set. The reference algorithm was traditional linear regression. The machine learning algorithms included random forest (RF), decision tree (DT), gradient boosting (GB), and multilayer perceptron (MLP).

The study constructed three predictive models by incorporating different variables. These models used patients’ demographic information, multimorbidity condition, and healthcare costs as inputs. Model A included only the prevalence of 29 multimorbidity conditions in the sample population, Model B added demographic characteristics such as patient age, gender, type of health insurance, and body mass index based on Model A, and Model C adds the patient’s 2018 health care costs based on Model B. The mean absolute error (MAE) and the mean absolute prediction error (MAPE) between the predicted value and actual value was used to assess the predictive performance, and the coefficient of determination R^2^ was used to reflect the regression fitting effect of the predictive model. Hyperparameter tuning involved five repetitions of 10-fold cross-validation and grid search. Finally, the impact of multimorbidity on healthcare costs was identified using SHapley Additive exPlanations (SHAP). The SHAP plot was adopted to visualize the contribution of the ten most important predictors from the best performing model. All analyses of predictive models were conducted using Python 3.0.

## Result

### Sample population characteristics

Table 1 presents the sociodemographic characteristics of 79,910 patients with type 2 diabetes in 2014. In the sample population, 85.76% of the diabetic patients were over 60 years old. The prevalence of obesity was 50.03% and males are more prevalent than females (52.27% vs. 47.89%). Approximately 82% of patients were enrolled in UEBMI and 18% were enrolled in URRBMI.

**Table 1:** Demographic characteristics of diabetic patients in China, 2014

Variable	Overall (*n* = 79,910)	Female (*n* = 40,899)	Male (*n* = 39,011)
Age group			
< 50	2,322(2.91)	905(2.21)	1,417(3.63)
50–59	9,056(11.33)	3,617(8.84)	5,439(13.94)
60–69	23,302(29.16)	11,504(28.13)	11,798(30.24)
70–79	25,449(31.85)	13,253(32.4)	12,196(31.26)
> 79	19,781(24.75)	11,620(28.41)	8,161(20.92)
BMI			
< 18.5	1,836(2.30)	1261(3.08)	575(1.47)
18.5–24	38,097(47.67)	20,051(49.03)	18,046(46.26)
> 24	39,977(50.03)	19,587(47.89)	20,390(52.27)
Insurance type			
UEBMI	65,667(82.18)	32,747(80.07)	32,920(84.39)
URRBMI	14,243(17.82)	8152(19.93)	6091(15.61)

### Prevalence of diabetes multimorbidity

In sample population, the number of diabetics with multimorbidity increased from 70,207 (87.9%) in 2014 to 79,320 (99.3%) in 2019. The average number of multimorbidity per person rose from 2.7 in 2014 to 7.4 in 2019 (Figure S1 in Online Supplementary Document). The number of multimorbidity conditions with prevalence increased more than 30% from 2014 to 2019 include arthritis (41.66%), chronic rhinitis/sinusitis/nasopharyngitis (35.65%), chronic gastritis (33.46%), chronic ischemic heart disease (31.79%), hyperlipidemia (31.38%), and chronic bronchitis (30.51%).

**Fig. 2:**
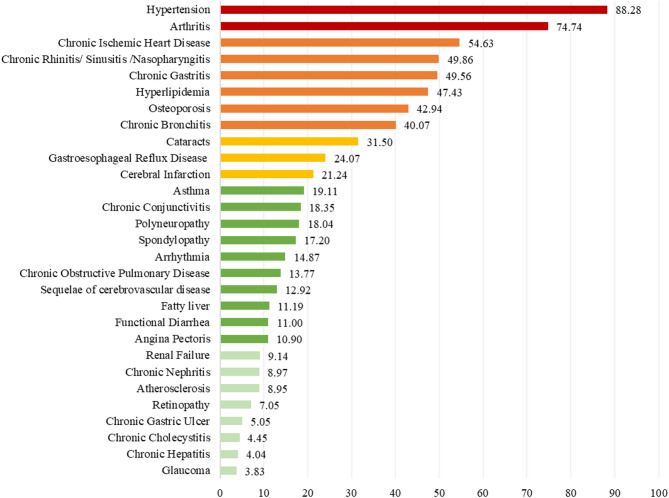
Prevalence of multimorbidity among diabetic patients in China, 2019 (%)

Among the 29 multimorbidity conditions, hypertension was the most common multimorbidity throughout the study period, with prevalence increasing from 69.58% in 2014 to 88.3% in 2019 (Fig. 2). In 2019, the prevalence of multimorbidity in people with diabetes exceeded 40%, including arthritis (74.7%), chronic ischemic heart disease (54.6%), chronic rhinitis/sinusitis/nasopharyngitis (49.9%), chronic gastritis (49.6%), hyperlipidemia (47.4%), osteoporosis (42.9%), and chronic bronchitis (40.07%). For detailed information on the change in prevalence of the 29 multimorbidity conditions from 2014 to 2019, please refer to Table S1 in Online Supplementary Document.

### Costs of diabetes multimorbidity

Between 2014 and 2019, the proportion of healthcare costs attributable to multimorbidity in diabetes increased from 31.8% to 34.2%. During this time, the average healthcare cost of multimorbidity rose from $903.2 to $1,674.2 (Figure S2 in Online Supplementary Document). The annual healthcare cost per capita associated with multimorbidity increased more than $500 from 2014 to 2019 include sequelae of cerebrovascular disease ($2873.4), cerebral infarction ($1946.9), chronic obstructive pulmonary disease ($992.4), angina pectoris ($637.9), and atherosclerosis ($516.1).

**Fig. 3:**
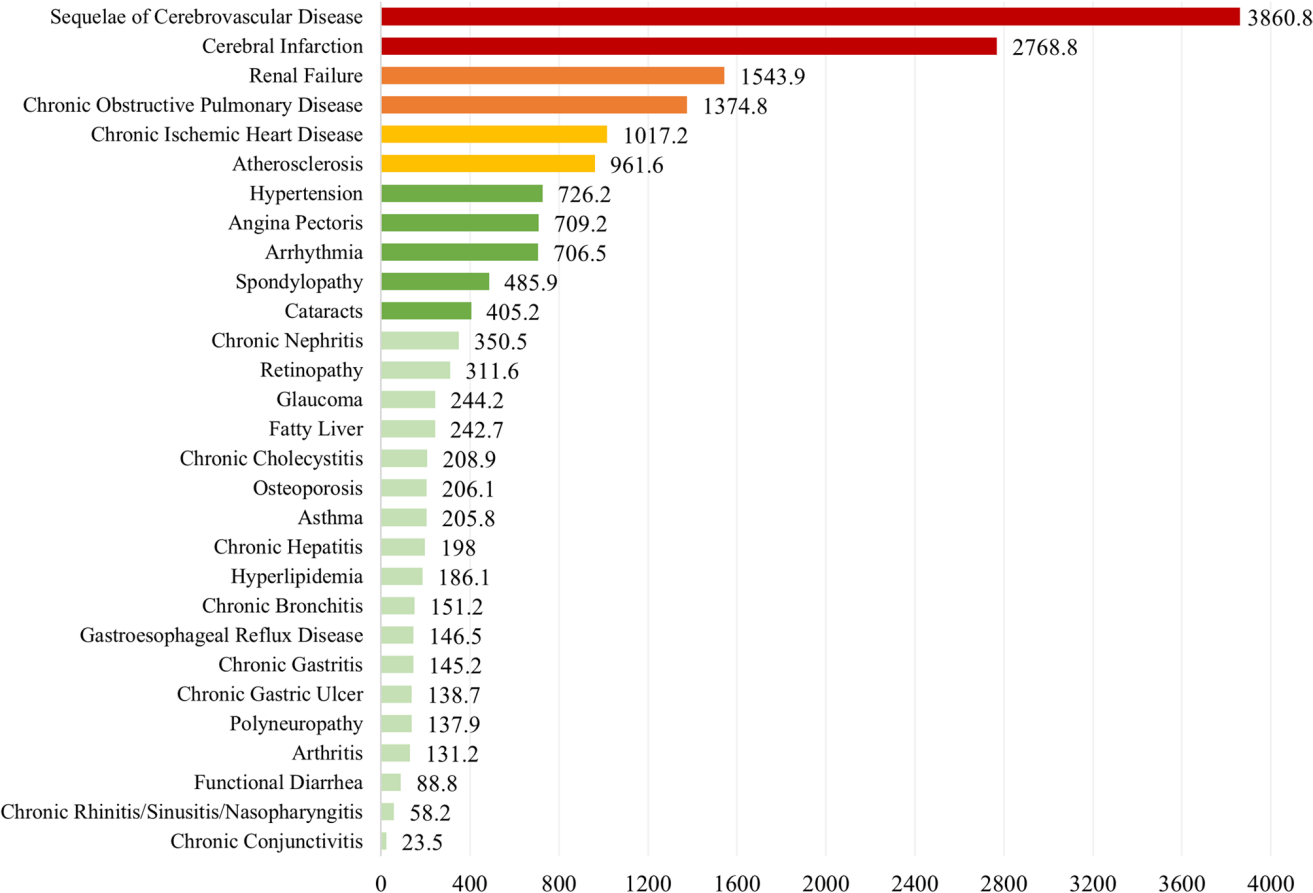
Per capita healthcare costs of multimorbidity among diabetic patients in China, 2019 ($)

In 2019, the conditions with the highest average medical costs were sequelae of cerebrovascular disease ($3,860.8), cerebral infarction ($2,768.8), renal failure ($1,543.9), chronic obstructive pulmonary disease ($1,374.8), and chronic ischemic heart disease ($1,017.2) (Fig. 3). For a detailed breakdown of the changes in the economic burden of the 29 multimorbidity conditions from 2014 to 2019, please refer to Table S2 in Online Supplementary Document.

### Predictive modeling of costs for diabetic patients

Table 2 shows the prediction performance of traditional linear regression and four machine learning algorithms under different models. The analysis showed that the machine learning algorithm outperforms traditional linear regression in terms of predictive performance, regardless of which model is used. When only multimorbidity conditions was used as a predictor variable (Model A), the R^2^ values of all algorithms ranged from 0.07 to 0.11. After adding demographic information (Model B), there was a slight improvement in predictive performance for all algorithms (R^2^: 0.11–0.20). With the inclusion of 2018 healthcare costs (Model C), the predictive performance of all algorithms substantially improved (R^2^: 0.50–0.58). Among these, the gradient boosting model still exhibited the best performance in terms of R^2^ (0.58), MAE (2777.37), and MAPE (3.54). Thus, the XGB algorithm and Model 3 were chosen to construct the final prediction model.

**Table 2:** Performance of different prediction models for the 2019 annual healthcare costs of diabetic patients in China

Model	Fit	Linear (Ref)	RF	DT	GB	MLP
A: Multimorbidity conditions	R^2^	0.07	0.09	0.08	0.11	0.09
	MAE	4777.60	4729.24	4738.78	4645.61	9565.87
	MAPE	5.35	5.17	5.20	4.94	5.09
B: Demo + Multimorbidity conditions	R^2^	0.11	0.14	0.13	0.20	0.16
	MAE	4417.00	4288.33	4318.06	4125.93	4200.45
	MAPE	6.09	5.97	5.99	5.62	5.94
C: Demo + Multimorbidity conditions + Cost 2018	R^2^	0.50	0.53	0.52	0.58	0.52
	MAE	2933.62	2856.30	2892.48	2777.37	2855.28
	MAPE	3.72	3.57	3.54	3.54	3.66

### Feature importance

**Fig. 4:**
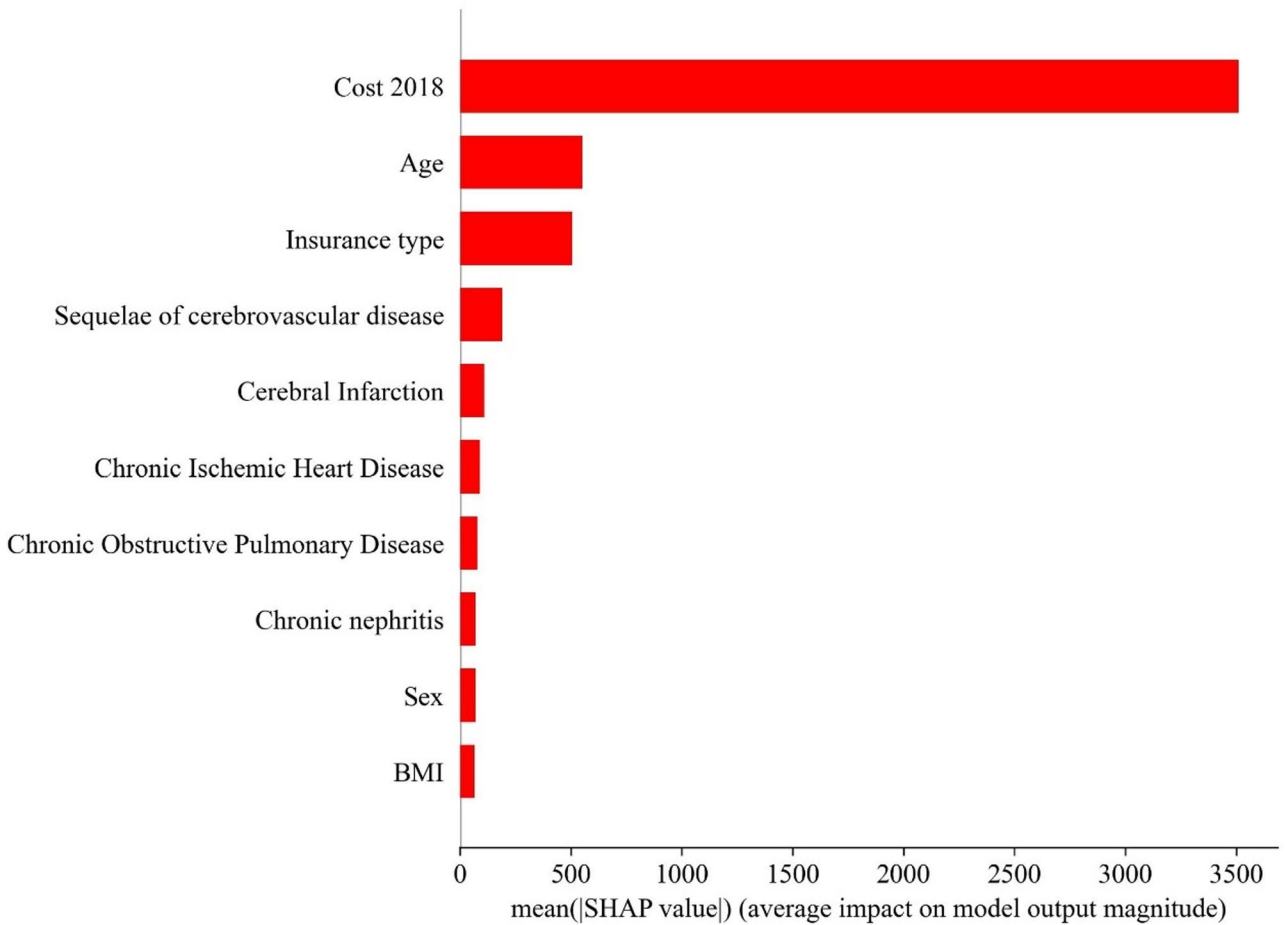
Feature importance in prediction models for the 2019 annual healthcare costs of diabetic patients in China

We performed SHAP analysis on the best predictive performer, Model 3 and the GB model, to understand the impact of multimorbidity on healthcare costs for type 2 diabetic patients. The SHAP values measured the marginal contribution of each variable to the predicted probability of annual healthcare costs for each individual, and this contribution was correlated with the values of the other variables. Variables with higher SHAP values were more likely to be associated with high-cost diabetes patients, and Fig. 4 shows the top 10 features in descending order of feature importance. The analysis showed that the variable that had the greatest impact on patients’ healthcare costs was the previous year’s healthcare costs. In terms of multimorbidity condition, sequelae of cerebrovascular disease, heart failure, chronic ischemic heart disease, chronic obstructive pulmonary disease, and chronic nephritis were important influences. Other important factors also included insurance type, sex, and BMI.

## Discussion

To the best of our knowledge, there is no similar study that comprehensively reflects the impact of multimorbidity on healthcare costs in China. Therefore, this study is the first to provide the prevalence and cost of 29 multimorbidities and use machine learning to determine the impact of multimorbidity on healthcare costs for people with diabetes. The analysis revealed that nearly 90% of people with diabetes have at least one multimorbidity, and the medical costs of multimorbidity account for more than 30% of the total medical costs. Among type 2 diabetes patients, the three most common multimorbidities were hypertension, arthritis, and chronic ischemic heart disease, while the most financially burdensome were sequelae of cerebrovascular disease, cerebral infarction, and renal failure. The multimorbidity that have important impact on future healthcare costs for patients with diabetes are sequelae of cerebrovascular disease, heart failure, chronic ischemic heart disease, chronic obstructive pulmonary disease, and chronic nephritis, and this impact is also related to the patient’s age, sex, insurance type, BMI, and prior annual healthcare costs.

Consistent with previous research, over 90% of diabetic patients have multimorbidity [19]. A study from the UK found that hypertension is the most common multimorbidity in diabetic patients, with a prevalence of 69.0%, which is in line with the baseline data of this study [20]. Additionally, a systematic literature review and Danish data reveals that the incidence of arthritis is over 40% and ischemic heart disease is over 20% among diabetic patients, further supporting the findings of this study [21, 22]. More important, prior research shown that modifiable risk factors—including smoking, poor diet, high BMI and physical inactivity—play a central role in the development of multimorbidity [23–25]. These factors not only increase disease burden but also contribute to disparities in healthcare utilization and costs. Therefore, individuals with diabetes should focus on managing their diet, exercise and lifestyle to prevent the development of multimorbidity and its economic impact [26]. However, it’s important to note that psychological disorders like depression and anxiety are also prevalent in diabetic patients, although this study did not analyze these conditions [27, 28]. This highlights the need for future research to better understand the impact of mental health on individuals with diabetes.

The results of the study showed that cardiovascular and renal diseases were the most burdensome multimorbidities for diabetic patients [29, 30]. When comparing the medical costs of diabetes multimorbidity in this study with another study in China, the average medical costs were lower in this study (ischemic heart disease: $1150 vs. $611, renal disease $1692 vs. $1332) [15]. This difference may be due to the different composition of the samples, with the urban population being the main body of this study, whereas the comparison study was based on the rural population. Furthermore, this study also found that the healthcare costs of diabetic patients with multimorbidity accounted for more than one-third of the total healthcare costs. Importantly, the economic impact rises significantly with the number and severity of multimorbidity [31, 32]. Therefore, prevention and control of the onset and progression of diabetes mellitus multimorbidity will be an important economic and clinical goal.

In addition, this study, like previous studies, demonstrated that machine learning models have a clear advantage over traditional regression models in predicting patient healthcare costs [33, 34]. For instance, studies from France and China compared the performance of machine learning model with linear regression model in predicting total, inpatient, and outpatient costs at the individual level [35, 36]. The results showed that the machine learning model outperformed the linear regression model. Furthermore, the machine learning model developed in this study showed improved prediction performance compared to previously studied machine learning models [35, 36].

More importantly, the study found having cardiovascular diseases or chronic obstructive pulmonary disease significantly impacts future healthcare costs for diabetic patients. Cardiovascular disease and chronic obstructive pulmonary disease not only increase the cost of healthcare for diabetic patients by more than 50%, but are important clinical features of high-cost, high-need patients [37–39]. Given the increasing global burden of cardiovascular and respiratory diseases, it is essential to devise new healthcare models for people with diabetes to mitigate these multimorbidities. The analysis highlights that a patient’s healthcare costs from the previous year are the most influential factor, possibly due to significant temporal correlations in individual patients’ healthcare expenditures [40]. Additionally, age, sex, insurance type, and BMI are key determinants of patients’ healthcare costs. Age, sex, and BMI are linked to the occurrence of multimorbidity, while insurance type impacts patients’ medical behavior [41, 42]. For example, individuals with lower income, education, or insurance are more likely to develop multimorbidity and experience higher healthcare costs due to delayed diagnosis and limited access to preventive care [42–44]. Therefore, the government should strengthen resource allocation for socioeconomically disadvantaged groups, particularly in less developed regions.

Based on these findings, clinicians should routinely conduct blood pressure monitoring, renal function assessment, and cardiovascular risk stratification during diabetes follow-up visits to ensure early detection and intervention for high-risk complications. For patients with a history of stroke or cardiovascular disease, multidisciplinary care should be strengthened and combined with lifestyle interventions and rehabilitation programs to reduce complications and hospital readmissions. Policymakers could prioritize insurance coverage for rehabilitation services and cost-effective medications, support community-based chronic disease management, health education, and telemedicine follow-up, and leverage predictive models to allocate resources toward high-risk populations and lower socioeconomic status groups. At the same time, reforms such as capitation and bundled payment should be promoted to both improve patient outcomes and control overall healthcare expenditures.

This study used a machine learning approach to analyze the impact of multimorbidity on healthcare costs for diabetic patients and to describe trends in multimorbidity prevalence and costs. This will help to complement the existing evidence and provide a basis for early prevention, clinical care and health management for people with type 2 diabetes. However, the study still has some limitations. First, the study population was drawn from developed cities in China, where healthcare accessibility and economic levels are relatively high. As a result, the findings may underestimate the challenges faced by rural and underdeveloped regions. Second, although missing values were excluded prior to analysis, the claims data may be subject to misclassification and lack important clinical details as well as data on social determinants of health. Third, although the inclusion of the previous year’s costs improves the model’s predictive performance, it may also lead to an underestimation of the impact of specific multimorbidity on healthcare expenses. Future research should utilize more diverse datasets to strengthen the evidence base and the representativeness of the results. Furthermore, although we used machine learning to compensate for potential shortcomings of traditional analytical methods, SHAP analysis can only provide the likelihood contribution of each variable in the model and still cannot offer information on exact effect sizes. Finally, this study was not comprehensive enough to investigate multimorbidity in diabetes. Future work should include a broader range of multimorbid conditions and explore the underlying relationships among different multimorbidity patterns.

## Conclusion

Multimorbidity conditions are common among diabetic patients and impose a significant economic burden. In particular, robust treatments and interventions are needed for the cerebrovascular disease sequelae, heart failure, chronic ischemic heart disease and chronic obstructive pulmonary disease. However, further studies with larger sample sizes are needed to fully understand the intrinsic relationship between diabetes multimorbidity.

## Supplementary Information

Supplementary Material 1.

## Data Availability

The datasets generated during and/or analyzed during the current study are not publicly available due to data confidentiality agreements with the sample cities but are available from the corresponding author (zhangluying@fudan.edu.cn) upon reasonable request.
