# Supplementary material for: Impact of multimorbidity on healthcare costs in patients with type 2 diabetes in China: a longitudinal analysis of health insurance claims data
Source: Arch Public Health. 2025 Nov 6;83:267. doi: 10.1186/s13690-025-01746-6 (PMC12590856; doi:10.1186/s13690-025-01746-6)

**Online Supplementary Document**

**Table of Contents**

[**Table S1: Prevalence of multimorbidity** **among diabetic patients in China from 2014 to 2019** 2](#_Toc210229679)

[**Table S2: Per capita healthcare costs of multimorbidity among diabetic patients in China from 2014 to 2019** 3](#_Toc210229680)

[**Figure S1: Number of multimorbidity among diabetic patients in China, 2014-2019** 4](#_Toc210229681)

[**Figure S2: Percentage of multimorbidity costs among diabetic patients in China, 2014-2019** 4](#_Toc210229682)

**Table S1: Prevalence of multimorbidity** **among diabetic patients in China from 2014 to 2019**

| Multimorbidity | 2014 | 2015 | 2016 | 2017 | 2018 | 2019 |
| --- | --- | --- | --- | --- | --- | --- |
| Hypertension | 69.58% | 76.64% | 81.02% | 84.29% | 86.65% | 88.28% |
| Arthritis | 33.08% | 48.48% | 58.24% | 65.38% | 70.93% | 74.74% |
| Chronic Ischemic Heart Disease | 22.84% | 32.69% | 40.00% | 45.85% | 50.49% | 54.63% |
| Chronic Rhinitis/ Sinusitis /Nasopharyngitis | 14.21% | 24.56% | 33.22% | 40.25% | 45.97% | 49.86% |
| Chronic Gastritis | 16.10% | 26.15% | 33.45% | 39.84% | 45.37% | 49.56% |
| Hyperlipidemia | 16.05% | 24.37% | 31.23% | 37.39% | 42.78% | 47.43% |
| Osteoporosis | 16.05% | 24.70% | 30.84% | 35.77% | 39.87% | 42.94% |
| Chronic Bronchitis | 9.56% | 18.02% | 25.14% | 30.82% | 36.19% | 40.07% |
| Cataracts | 9.48% | 15.42% | 20.67% | 24.93% | 28.58% | 31.50% |
| Gastroesophageal Reflux Disease | 6.16% | 11.03% | 14.75% | 18.00% | 21.34% | 24.07% |
| Cerebral Infarction | 7.37% | 10.91% | 13.88% | 16.73% | 18.87% | 21.24% |
| Asthma | 5.11% | 8.69% | 11.64% | 14.29% | 16.91% | 19.11% |
| Chronic Conjunctivitis | 2.76% | 5.65% | 8.81% | 12.26% | 15.65% | 18.35% |
| Polyneuropathy | 3.96% | 7.07% | 10.00% | 12.79% | 15.53% | 18.04% |
| Spondylopathy | 3.79% | 7.02% | 10.38% | 13.08% | 15.32% | 17.20% |
| Arrhythmia | 4.11% | 6.89% | 9.30% | 11.43% | 13.33% | 14.87% |
| Chronic Obstructive Pulmonary Disease | 3.88% | 6.68% | 8.75% | 10.58% | 12.27% | 13.77% |
| Sequelae of cerebrovascular disease | 3.26% | 5.55% | 7.56% | 9.50% | 11.04% | 12.92% |
| Fatty liver | 3.39% | 5.90% | 7.70% | 9.08% | 10.30% | 11.19% |
| Functional Diarrhea | 1.61% | 3.42% | 5.23% | 7.11% | 9.07% | 11.00% |
| Angina Pectoris | 4.07% | 6.37% | 7.77% | 9.00% | 9.93% | 10.90% |
| Renal Failure | 1.90% | 3.06% | 4.40% | 5.85% | 7.56% | 9.14% |
| Chronic Nephritis | 2.39% | 3.69% | 4.89% | 6.19% | 7.59% | 8.97% |
| Atherosclerosis | 1.41% | 2.78% | 4.26% | 5.90% | 7.42% | 8.95% |
| Retinopathy | 1.84% | 3.17% | 4.28% | 5.25% | 6.18% | 7.05% |
| Chronic Gastric Ulcer | 1.25% | 2.24% | 3.01% | 3.72% | 4.45% | 5.05% |
| Chronic Cholecystitis | 1.06% | 1.76% | 2.55% | 3.28% | 3.89% | 4.45% |
| Chronic Hepatitis | 1.45% | 2.26% | 2.77% | 3.23% | 3.65% | 4.04% |
| Glaucoma | 1.20% | 1.82% | 2.36% | 2.86% | 3.40% | 3.83% |

**Table S2: Per capita healthcare costs of multimorbidity among diabetic patients in China from 2014 to 2019**

| Multimorbidity | 2014 | 2015 | 2016 | 2017 | 2018 | 2019 |
| --- | --- | --- | --- | --- | --- | --- |
| Sequelae of Cerebrovascular Disease | 987.4 | 1246.7 | 2011.8 | 2776.7 | 3081.9 | 3860.8 |
| Cerebral Infarction | 821.9 | 1092.1 | 1379.7 | 1751.6 | 2174.1 | 2768.8 |
| Renal Failure | 1286.2 | 1332.0 | 1230.9 | 1357.5 | 1264.6 | 1543.9 |
| Chronic Obstructive Pulmonary Disease | 382.4 | 429.3 | 513.2 | 734.3 | 921.6 | 1374.8 |
| Chronic Ischemic Heart Disease | 546.8 | 611.2 | 690.1 | 824.3 | 858.6 | 1017.2 |
| Atherosclerosis | 445.5 | 479.0 | 640.5 | 709.5 | 856.2 | 961.6 |
| Hypertension | 467.2 | 501.5 | 540.5 | 612.0 | 675.8 | 726.2 |
| Angina Pectoris | 71.3 | 111.2 | 124.3 | 405.0 | 714.2 | 709.2 |
| Arrhythmia | 311.8 | 397.3 | 399.2 | 470.8 | 545.6 | 706.5 |
| Spondylopathy | 259.2 | 341.1 | 325.4 | 280.3 | 356.9 | 485.9 |
| Cataracts | 239.7 | 273.9 | 289.6 | 310.4 | 351.1 | 405.2 |
| Chronic Nephritis | 243.6 | 299.3 | 283.2 | 295.8 | 308.2 | 350.5 |
| Retinopathy | 181.5 | 206.8 | 241.6 | 291.8 | 306.1 | 311.6 |
| Glaucoma | 202.0 | 216.9 | 215.7 | 214.0 | 195.3 | 244.2 |
| Fatty Liver | 169.7 | 142.8 | 149.7 | 153.4 | 229.6 | 242.7 |
| Chronic Cholecystitis | 87.7 | 117.6 | 136.0 | 212.4 | 161.0 | 208.9 |
| Osteoporosis | 121.2 | 140.1 | 151.8 | 164.3 | 204.3 | 206.1 |
| Asthma | 135.2 | 124.9 | 146.2 | 151.0 | 176.4 | 205.8 |
| Chronic Hepatitis | 168.8 | 196.5 | 181.8 | 179.0 | 173.7 | 198.0 |
| Hyperlipidemia | 152.8 | 162.3 | 164.4 | 172.1 | 181.7 | 186.1 |
| Chronic Bronchitis | 93.3 | 89.1 | 96.3 | 99.4 | 126.5 | 151.2 |
| Gastroesophageal Reflux Disease | 94.6 | 105.6 | 103.9 | 127.5 | 139.1 | 146.5 |
| Chronic Gastritis | 106.2 | 118.9 | 124.8 | 138.9 | 155.8 | 145.2 |
| Chronic Gastric Ulcer | 97.8 | 97.0 | 91.0 | 131.8 | 194.4 | 138.7 |
| Polyneuropathy | 91.8 | 96.8 | 111.4 | 119.6 | 120.5 | 137.9 |
| Arthritis | 89.6 | 102.7 | 108.4 | 117.6 | 126.9 | 131.2 |
| Functional Diarrhea | 56.2 | 59.4 | 70.1 | 77.5 | 81.7 | 88.8 |
| Chronic Rhinitis/Sinusitis/Nasopharyngitis | 43.1 | 45.4 | 56.3 | 70.6 | 71.0 | 58.2 |
| Chronic Conjunctivitis | 16.6 | 17.4 | 18.5 | 20.8 | 22.5 | 23.5 |

**Figure S1: Number of multimorbidity among diabetic patients in China, 2014-2019**


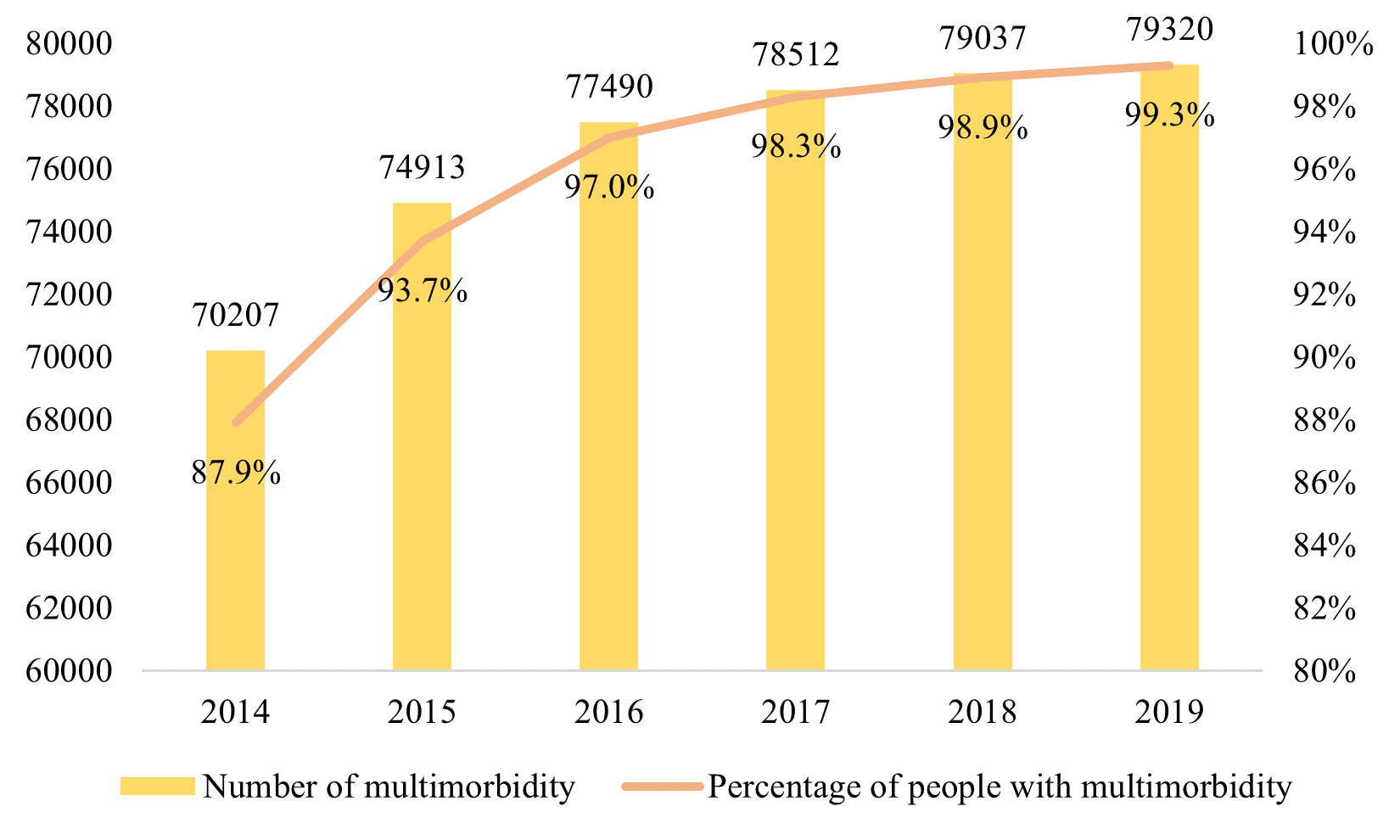


**Figure S2: Percentage of multimorbidity costs among diabetic patients in China, 2014-2019**


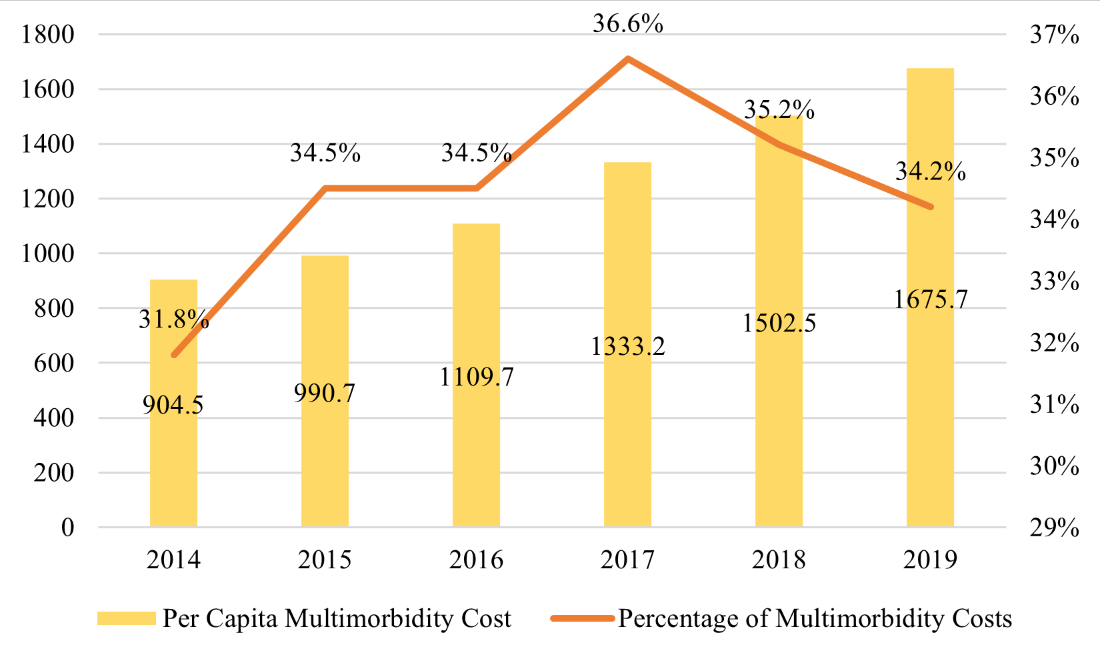

Supplement: Supplementary file 1 — Supplementary Material 1. [file 13690_2025_1746_MOESM1_ESM.docx]
